# SRVF, a novel herbal formula including *Scrophulariae Radix* and *Viticis Fructus*, disrupts focal adhesion and causes detachment-induced apoptosis in malignant cancer cells

**DOI:** 10.1038/s41598-017-12934-y

**Published:** 2017-10-16

**Authors:** Aeyung Kim, Minju Im, Jin Yeul Ma

**Affiliations:** Korean Medicine (KM) Application Center, Korea Institute of Oriental Medicine (KIOM), 70 Chumdan-ro, Dong-gu, Daegu, 41062 Republic of Korea

## Abstract

When cells lose adhesion, they undergo detachment-induced apoptosis, known as anoikis. In contrast, tumor cells acquire resistance to anoikis, enabling them to survive, even after separating from neighboring cells or the ECM. Therefore, agents that restore anoikis sensitivity may serve as anti-cancer candidates. In this study, we constructed a novel herbal formula, SRVF, which contains *Scrophulariae Radix* (SR) and *Viticis Fructus* (VF). SRVF rapidly decreased cell adhesion, altered the cell morphology to round, and induced cell death; however, SR, VF, or their co-treatment did not. SRVF arrested HT1080 cells in G_2_/M phase, increased the levels of pro-apoptotic proteins, and decreased the levels of anti-apoptotic proteins. Furthermore, SRVF efficiently reduced cell-cell and cell-ECM interactions by disrupting the F-actin cytoskeleton and down-regulating the levels of focal adhesion-related proteins, suggesting that SRVF efficiently triggers detachment-induced apoptosis (i.e., anoikis) in malignant cancer cells. In xenograft mouse models, daily oral administration of 50 or 100 mg/kg SRVF retarded tumor growth *in vivo*, and repeated administration of SRVF did not cause systemic toxicity in normal mice. These data collectively indicate that SRVF induces cancer cell death by restoring anoikis sensitivity *via* disrupting focal adhesion. Therefore, SRVF may be a safe and potent anti-cancer herbal decoction.

## Introduction

Cell-extracellular matrix (ECM) adhesion plays an essential role in several cellular functions such as differentiation, proliferation, and motility^1^. Normal epithelial cells adhere to the ECM through interactions between specific integrin receptors and the ECM counterparts^2^. When cells lose cell-ECM adhesion or relocate to an inappropriate environment, they rapidly undergo programmed cell death referred to anoikis, a Greek word meaning “homelessness”^3^. Anoikis inhibits detached cells’ reattachment to a new inadequate ECM and their dysplastic growth, thus acting as a self-defense barrier against oncogenic transformation by eliminating misplaced cells^3^. Anoikis is mediated by the interplay of two apoptotic pathways, namely, the intrinsic pathway (due to mitochondrial perturbation) and the extrinsic pathway (triggered by the cell surface death receptor). In the intrinsic pathway, pro-apoptotic proteins including Bax/Bak are activated, while the anti-apoptotic functions of Bcl-2 are suppressed by BH3-only proteins. Thereafter, mitochondrial cytochrome c is released into the cytoplasm and caspases are activated. In the extrinsic pathway, engagement of the death receptor induces caspase-8 activation, leading to the cleavage and activation of caspases, endonuclease activation, DNA fragmentation and, ultimately, cell death^4,5^.

The dysregulation of anoikis, known as “anoikis resistance”, is an important hallmark of cancer cells, particularly in the process of tumor metastasis^4,6^. Cancer cells that are resistant to anoikis can survive even after detachment from the ECM at the primary site, migrate through the systemic circulation, and colonize at a secondary site, leading to metastasis^7,8^. Anoikis resistance has been characterized in many types of human malignancies, including gastric, colon, lung, breast, and ovarian cancers, and is a prerequisite for metastases, the major cause of cancer mortality. It has been reported that the epithelial-mesenchymal transition (EMT) accompanied by the loss of E-cadherin and the induction of N-cadherin *via* the expression of Snail, Twist, and Zeb1 is an essential process for anoikis resistance^9^. In addition, overexpression of growth factor receptors, such as hepatocyte growth factor (HGF) receptor and epidermal growth factor (EGF) receptor, and oncogenes, such as ErbB family members, suppress anoikis by activating pro-survival signaling pathways such as PI3k/Akt, Ras/MAPK, NF-κB, Rho-GTPase, and STAT3. Focal adhesions are specialized complexes formed at the connection between cells and the ECM, and contribute to various physiological processes, such as proliferation, survival, migration, and differentiation. Focal adhesion kinase (FAK) is overexpressed in cancer cells and is critical for anoikis resistance^4,10^. Therefore, overcoming anoikis resistance or restoring anoikis sensitivity may represent potential strategies for the prevention of outgrowth and metastasis of cancer cells. Anoikis-sensitizing agents elicit anti-proliferative and anti-invasive activities against malignant cancer cells by suppressing the FAK and EMT pathways, as well as caspase activation.

In previous studies, herbal cocktails (multi-herb mixtures extracted in a single formula) have shown a much stronger therapeutic efficacy than when used individually as each herbal component or when used in co-treatment, while alleviating the toxic side effects^11,12^. In this study, we formulated a novel herbal cocktail, SRVF, which is composed of *Scrophulariae Radix* (SR) and *Viticis Fructus* (VF). SR is a perennial plant that grows in all parts of Korea, China, and Asia, and has been used as a folk medicine as an anti-phlogistic, anti-pyretic, and analgesic. Since SR can remove heat, cool the blood, nourish yin, and relieve toxins, it is commonly used in combination with other herbs as a nutrient and as a health strengthening agent. Moreover, SR was recently demonstrated to exhibit anti-oxidant, anti-inflammatory, anti-depressant, anti-dementia, anti-diabetic, anti-pruritic, anti-hepatotoxic, and anti-neurotoxic activities^13–15^. VF is a dried ripened fruit of *Vitex rotundifolia*, which is widely distributed along the coast in Asian countries, and has been used to treat headaches, colds, migraines, eye pain, asthma, chronic bronchitis, and gastrointestinal infections for many years as a folk medicine^16^. Casticin, an active component of VF, exhibits anti-nociceptive and anti-hyperprolactinemia activities, and is thus effective in the treatment of pre-menstrual symptoms^17,18^. In addition, casticin and vitexicarpin, isolated from VF, induce apoptosis of various cancer cells through the generation of reactive oxygen species (ROS), endoplasmic reticulum stress, or G_2_/M cell cycle arrest^19–21^.

In the present study, we examined the effect of SRVF on the induction of cell death in malignant cancer cells and elucidated the detailed mechanisms of its anti-cancer activity. In addition, to demonstrate the superiority of the herbal cocktail, the anti-cancer activity of SRVF was compared to that of SR, VF, and their co-treatment. Furthermore, *in vivo* tumor growth inhibition by oral administration of SRVF was examined using a xenograft mouse model.

## Materials and Methods

### Cell culture and mice

Human breast carcinoma MDA-MB-231 cells (ATCC HTB-26), human hepatocellular carcinoma HepG2 cells (ATCC HB-8065), human normal fibroblast cells Hs27 (ATCC CRL-1634), and human normal mammary epithelial cells MCF-10A (ATCC CRL-10317) were all purchased from American Type Culture Conditions (ATCC, Rockville, MD). Human fibrosarcoma HT1080 cells (KCLB No. 10121) was purchased from Korean Cell Line Bank (Seoul, Korea). Cells were maintained with Dulbecco’s modified Eagle’s medium (DMEM) or Roswell Park Memorial Institute (RPMI) 1640 medium (Lonza, Walkerswille, MD, USA) containing 10% fetal bovine serum (FBS; Biotechnics Research, Lake Forest, CA, USA) and 1% penicillin/streptomycin (Cellgro, Manassas, VA, USA) in a humidified incubator with 5% CO_2_. For xenograft model, female Balb/c nude mice (5 to 6 weeks old, weighing approximately 16 to 18 g) were obtained from Orient Bio (Sungnam, Korea). The mice (5 mice/cage) were housed in a specific-pathogen-free facility under 12 h light and dark cycle at 22 ± 1 °C and 55 ± 5% humidity and fed standard chow diet with water ad libitum. Animal experiment (reference numbers #D-17-003 and #D-17-003-1) approved by the Animal Care and Use Committee of the Korea Institute of Oriental Medicine (KIOM, Daegu, Korea) was performed in accordance with their guidelines.

### Reagents and antibodies

Propidium iodide (PI), 4′,6-Diamidino-2-phenylindole dihydrochloride (DAPI), and TRITC-labeled phalloidin from *Amanita phalloides* were purchased from Sigma Chemical Co. (St. Louis, MO, USA). Antibodies against p21, p27, cyclin B, cyclin D, cyclin E, CDK2, CDK4, CDK6, Bax, Bcl-2, caspase-3, poly ADP ribose polymerase (PARP), p38, p-p38 (Thr180/Tyr182), extracellular regulated kinase (ERK)1/2, p-ERK1/2 (Thr202/Tyr204), c-Jun N-terminal kinase (JNK), p-JNK (Thr183/Tyr185), STAT3, p-STAT3 (Tyr705), p-STAT3 (Ser727), and tubulin were obtained from Cell Signaling Technology (Danvers, MA, USA). Focal adhesion protein antibody kit including antibodies against α-Actinin, FAK, Paxillin, Talin-1, Tensin-2, and Vinculin was purchased from Cell Signaling Technology.

### Preparation of SR, VF, and SRVF

*Scrophulariae Radix* (SR) and *Viticis Fructus* (VF) were purchased as dried herbs from Yeoncheon Hyundai Herbal Market (Yeoncheon, Korea). After confirmation of the identity by Professor Ki Hwan Bae (Chungnam National University, Korea), herbs were stored at 4 °C in the herbal bank of the Korean Medicine (KM) Application Center (Daegu, Korea). To prepare water extracts of SR and VF, dried SR or VF (50 g) was soaked in distilled water (1 liter) and heat-extracted in a Cosmos-600 Extractor (Gyeonseo Co., Incheon, Korea) for 3 h at 115 °C. The extracts were filtered through standard testing sieves (150 μm, Retsch, Haan, Germany), freeze-dried, and then stored in a desiccators at 4 °C. Water extract of SRVF, a novel herbal formula containing SR and VF at the ratio of 3:2, were also heat-extracted as detailed above. The amount of collected SR, VF, and SRVF powder was 14.79 g, 2.07 g, and 4.735 g, therefore the yield was 29.58%, 4.14%, and 9.47%, respectively. For *in vitro* experiments, stock solutions were prepared by dissolving powder in 10% DMSO to a final concentration of 50 mg/ml. After centrifugation at 14,000 rpm for 15 min, the supernatant was collected, filtered using 0.22-µm disk filter, and then stored at −20 °C.

### Cell cytotoxicity assay

The cytotoxicity of SR, VF, and SRVF was measured using CCK assay kit (cell counting kit-8, Dojindo Laboratories, Kumamoto, Japan). In brief, cells were plated in 96-well culture plates (5,000 cells/well/100 µl), incubated for 12 h to allow the cells to attach to the plates, and then treated with indicated concentrations of SR, VF, and SRVF. After incubation for an appropriate length of time, 10 µl of CCK-8 solution was added to each well of the plate. Following incubation for 1–4 h, the absorbance was measured at 450 nm using a SpectraMaxi3 microplate reader (Molecular Devices, Sunnyvale, CA, USA). To generate colonies from single cell to several hundred cells, 2 ml of cell suspension was dispensed in 12-well culture plates at a density of 200 cells/well and incubated for 7–10 days in the complete culture medium. The culture medium was changed every two days.

### Cell cycle analysis

Cells treated with indicated concentrations of SR, VF, or SRVF for 12 h and 24 h were harvested, washed with PBS, and fixed in ice-cold 70% ethanol at −20 °C for overnight. The fixed cells were washed two times with PBS and stained with PI solution containing 0.1% Triton X-100, 0.1 mM EDTA, 50 μg/ml RNase A and 50 μg/ml PI in PBS at 4 °C for 30 min. Acquisition and analysis of cell cycle distribution data was performed on a Gallios flow cytometer and Kaluza software (Beckman Coulter, Inc. Brea, CA, USA), respectively.

### Live and dead cell staining

To visualize the live and dead cells, LIVE/DEAD Cell Imaging Kit (Invitrogen, Carlsbad, CA, USA) wa﻿s used. In brief, cells were seeded in 24-well culture plates, incubated for 12 h to allow cells to attach, and then replaced with fresh culture medium containing SR, VF, or SRVF at the indicated doses. After incubation for 24 h, equal volume of 2× working solution containing Live Green (component A) and Dead Red (component B) was added to cells, incubated for 15 min at 20–25 °C, and then imaged under a fluorescence microscope (Nikon Eclipse Ti, Nikon instruments, Kanagawa, Japan).

### Immunoblot analysis

Whole proteins were extracted with M-PER Mammalian Protein Extraction Reagent (Thermo Scientific, Rockford, IL, USA) and clarified by centrifugation at 12,000 rpm for 15 min at 4 °C. Protein concentration of lysates was determined using a BCA protein assay kit (Sigma, St. Louis, MO, USA) and lysates were denatured with SDS sample buffer followed by boiling. Same amount of protein aliquots were separated on SDS-PAGE and immunoblotted using specific antibodies. Proteins were visualized under a ChemiDoc^TM^ Touch Imaging System (Bio-Rad, Hercules, CA, USA) with Bio-Rad Clarity^TM^ Western ECL Substrate. The expression profile of 33 human apoptosis-related proteins in SRVF-treated or untreated cells was determined using a Proteome Profiler Human Apoptosis Array Kit (ARY009, R&D System, Minneapolis, MN, USA) according to the manufacturer’s protocol.

### Monitoring cell proliferation and adhesion

Cell proliferation and adhesion were monitored using the xCELLigence Real-Time Cell Analyzer (RTCA) system as per manufacturer’s instruction (Roche Applied Science, Mannheim, Germany). Cells (10,000 cell/well/100 µl) were seeded on E-Plate 16, allowed to attach onto the electrode surface, and then replaced with fresh medium containing SR, VF, or SRVF either from the beginning or 20 h post-seeding. The electrical impedance was automatically recorded every 15 min and expressed as a Cell Index (CI) value.

### Immunofluorescence staining

Cells grown to about 80% confluence on the coverslips in 24-well culture plates were incubated in the serum free medium for 6 h, treated with SR, VF, or SRVF, and further incubated for 6 h. After fixing with 2% formaldehyde in PBS for 10 min, cells were washed twice with washing buffer (10% FBS, 0.05% sodium azide in PBS) for 5 min. To visualize the filamentous actin, cells were stained with TRITC-labeled phalloidin diluted to 1:1000 in labeling buffer (10% FBS, 0.05% sodium azide, 0.2% saponin in PBS) for 1 h at room temperature. Cells were washed three times with washing buffer and the coverslips were mounted with VECTASHIELD (mounting medium with DAPI, Vector Laboratories, Burlingame, CA, USA).

### *In vivo* tumor growth inhibition assay

Six-week-age female Balb/c nude mice were subcutaneously injected into the abdominal region with HT1080 cells (2 × 10^6^ cell/200 µl PBS). On day 7 post-inoculation of tumor cell when tumor volume reached to about 50–100 mm^3^, mice were randomly divided into 6 groups (n = 6 per group) and daily administered with saline (control), SR, VF, SRVF, and SR + VF in a volume of 100 µl for 14 days. During experiments, mice were carefully observed for the appearance, motility, and behavior. In addition, tumor volume was measured twice per week. On day 21, tumors were excised and weighed after euthanasia by inhalation of isoflurane. To assess the safety of SR, VF, and SRVF, 6-week-old female Balb/c nude mice were divided into the 5 groups (n = 3 per group) and fed with vehicle (saline), SR, VF, or SRVF daily during 14 days. At the time of sacrifice, weights of major organs and body weight were measured. For the detection of proliferating cells in xenograft tumors, immunochemical staining for Ki67 was performed on 20 µm-thick cryosections using anti-Ki67 antibody (Abcam, Cambridge, MA, USA) and avidin-biotinylated enzyme complex (ABC) with DAB staining (Vectastatin Elite ABC kit, Vector DAB Substrate kit, Vector Laboratories, Burlingame, CA, USA).

### Statistics

Data were expressed as means ± standard deviation (SD) and statistical significance of mean values in two groups was analyzed by Student’s *t*-test. All variables were analyzed by GraphPad PRISM software (GraphPad PRISM software Inc., Version 5.03, CA, USA) and treatment effect was analyzed using one-way ANOVA by Dunnett’s multiple comparison test. A *p*-value less than 0.05 was considered as statistically significant.

## Results

### SRVF dramatically decreased cell viability of malignant cancer cells relative to SR, VF, or their co-treatment

**Figure 1:**
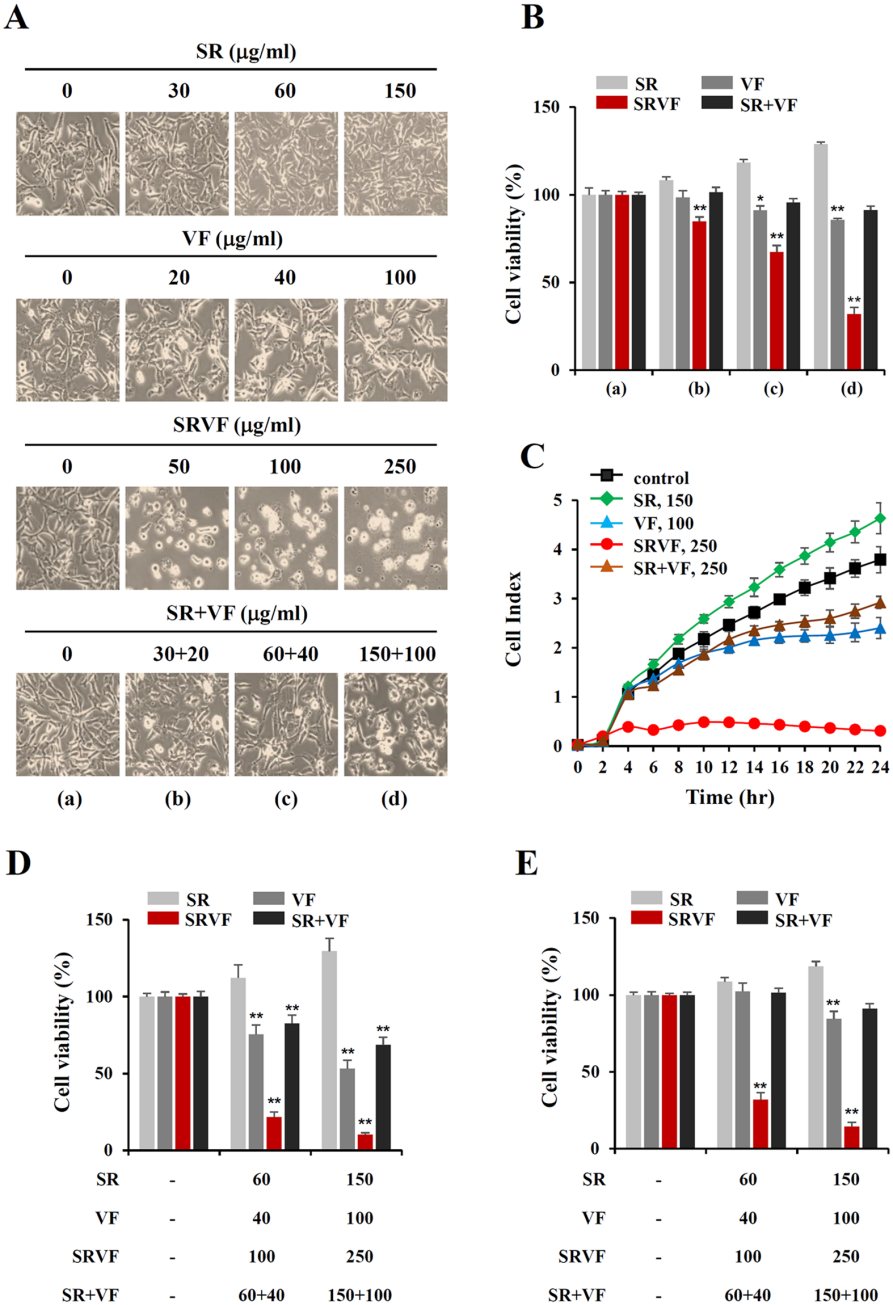
SRVF dramatically alters cell morphology and decreases cell viability in malignant cancer cells. (**A**) HT1080 cells were incubated with the indicated doses of SR, VF, or SRVF at concentrations of 50, 100, or 250 µg/ml for 24 h and photographed at a magnification of ×200 under an inverted microscope. (**B**) Cell viability after incubation with SR, VF, or SRVF was determined using the CCK assay and is expressed as the mean ± standard deviation (SD). (**C**) Cells were seeded onto the E-Plate 16, treated with SR, VF, or SRVF at a concentration of 250 µg/ml, and cell proliferation was monitored using the xCELLigence system. Data are representative of two independent experiments performed in triplicate and are expressed as the mean ± SD. (**D**,**E**) The effects of SR, VF, or SRVF on cell viability of MDA-MB231 (**D**) and HepG2 (**E**) cells were evaluated by the CCK assay and are expressed as the mean ± SD. Statistical significance was determined with Student’s *t*-test. *p < 0.05, **p < 0.05 vs. untreated control.

To identify a novel herbal decoction with potent anti-cancer activity, we screened several hundreds of herbal mixtures and examined one: SRVF. SRVF is an herbal decoction containing SR and VF at a ratio of 3:2. We first examined the anti-proliferative effect of SR and VF at concentrations of 50, 100, and 250 µg/ml SRVF. As shown in Fig. 1A,B, SR increased the proliferation of HT1080 cells in a dose dependent manner and reached the viability to approximately 130% of the untreated control cells at 150 µg/ml, whereas VF at 100 µg/ml slightly decreased cell proliferation to approximately 85% of the untreated control cells. Exposure to SRVF caused dramatic morphological changes, such as shrinkage and floating, which led to a reduction in cell viability to 84.9%, 67.4%, and 32% at 50, 100, and 250 µg/ml (F = 282.4, *p* < 0.0001, one-way ANOVA), respectively, whereas co-treatment with SR and VF at the same concentrations did not. Data obtained from the xCELLigence system, which represents the electrical impedance of cells on the basis of cell number, adhesion, and spreading^22^, also revealed the potent anti-proliferative activity of SRVF in HT1080 cells (Fig. 1C), indicating that SR and VF exert greater anti-proliferative activity when used together as an herbal cocktail rather than in combination. In other malignant cancer cells, including MDA-MB231 and HepG2 cells, SRVF efficiently decreased cell viability, surpassing the additive effects of SR and VF co-treatment (MDA-MB231 cells; F = 1534, *p* < 0.0001, HepG2 cells; F = 679.7, *p* < 0.0001, one-way ANOVA) (Fig. 1D,E). In contrast, normal cells (e.g., Hs27 and MCF-10A cells) were not affected by SRVF in terms of cell viability, indicating that SRVF is not cytotoxic to normal cells (Supplementary Fig. 1).

### SRVF induced G_2_/M cell cycle arrest and regulated the expression of cell cycle-related proteins

**Figure 2:**
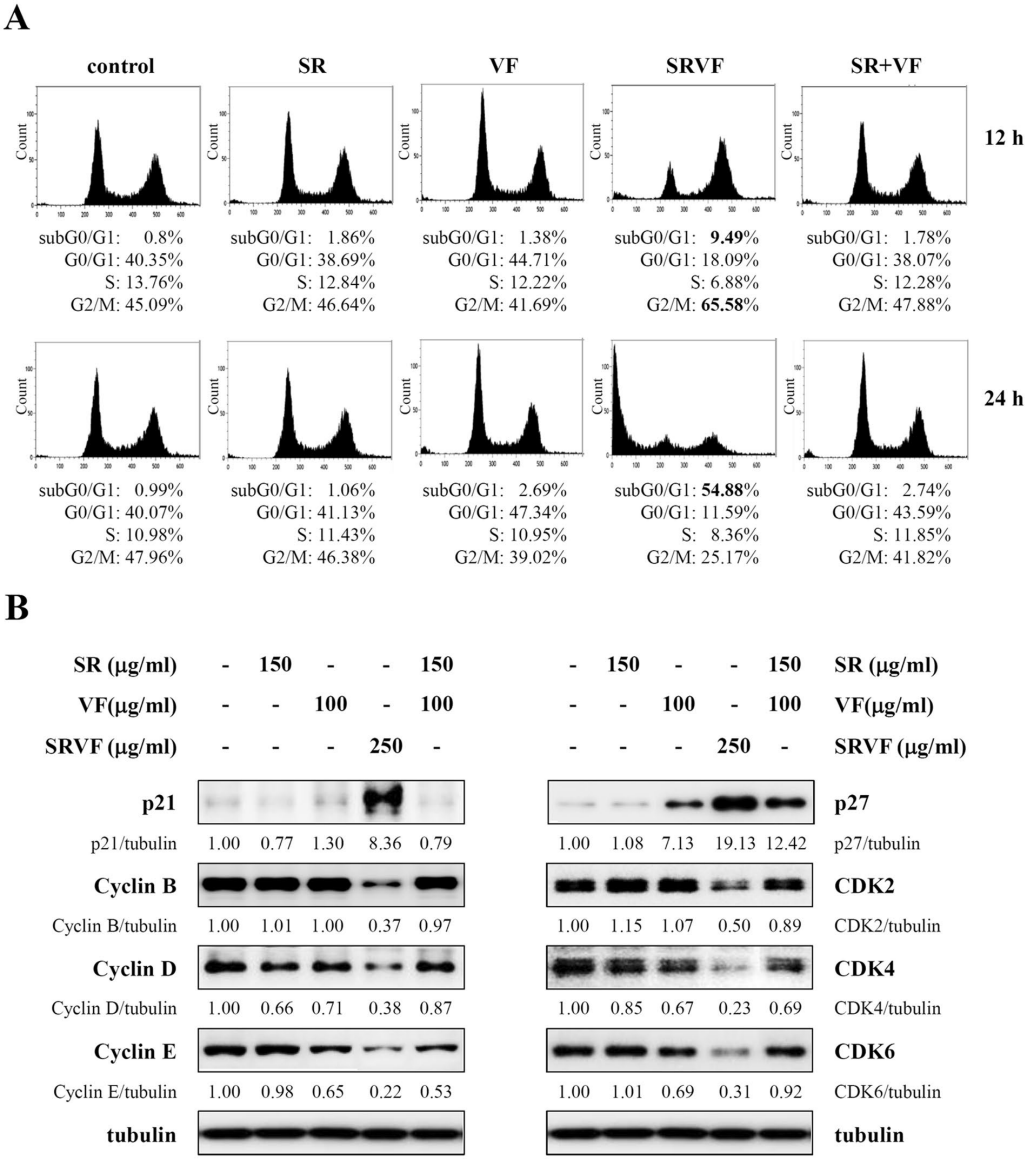
SRVF induces cell cycle arrest at G_2_/M phase in HT1080 cells. (**A**) Cells were incubated with SR, VF, or SRVF at a concentration of 250 µg/ml for 12 or 24 h, and cell cycle distribution was examined by flow cytometry after staining intracellular DNA with propidium iodide solution. Cell cycle distribution was analyzed using Kaluza software and is expressed as the relative percentage. Data are representative of three independent experiments. (**B**) The expression of cell cycle-related proteins was examined by Western blot analysis in cells treated with SR, VF, or SRVF at a concentration of 250 µg/ml for 12 h. Relative band intensities were calculated using ImageJ software after normalizing to tubulin expression. The full size blot is shown in Supplementary Fig. S2.

We next analyzed cell cycle distribution in HT1080 cells treated with SR, VF, or SRVF for 12 or 24 h at a concentration of 250 µg/ml. As shown in Fig. 2A, propidium iodide (PI) staining for intracellular DNA content revealed that SRVF treatment for 12 h increased the proportion of cells in G_2_/M phase to 65.58%, as compared to untreated control cells (45.09%); this was accompanied by a decrease in the proportion of cells in G_1_ and S phase. In addition, apoptotic cells in subG_0_/G_1_ phase were considerably increased to 9.45% and 54.88% by SRVF treatment for 12 and 24 h, respectively. In contrast, treatment with SR or VF alone or in combination did not significantly affect cell cycle progression, while VF treatment for 12 and 24 h slightly increased the proportion of cells in G1 phase to 44.71% and 47.34%, respectively, as compared to control cells (40.35% and 40.07%, respectively). Western blot analysis revealed that SRVF significantly up-regulated the levels of cyclin-dependent kinase (CDK) inhibitors p21 and p27, and down-regulated the levels of cyclin B, cyclin D, cyclin E, CDK2, CDK4, and CDK6 relative to untreated control cells (Fig. 2B). VF or co-treatment with SR and VF slightly altered the levels of cell cycle-related proteins; however the effect was much lower than that of SRVF.

### SRVF induced apoptosis more efficiently than SR, VF, or their co-treatment

**Figure 3:**
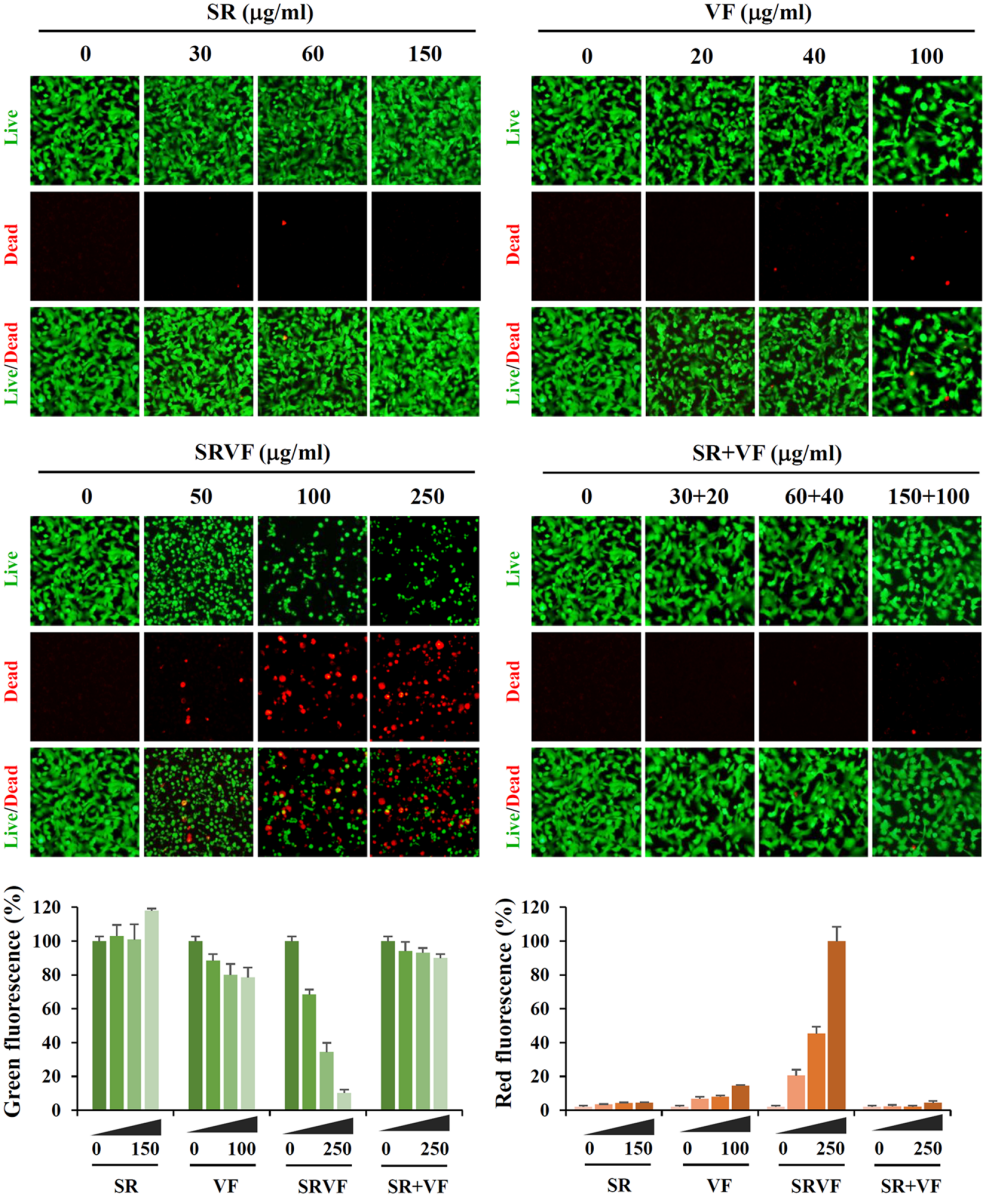
SRVF causes severe cytotoxicity in HT1080 cells. After incubating cells with the indicated concentrations of SR, VF, or SRVF for 24 h, live and dead cells were examined under a fluorescence microscope using the LIVE/DEAD Cell Imaging Kit. Data are expressed as the mean ± SD of five random fields per sample and are representative of three independent experiments.

**Figure 4:**
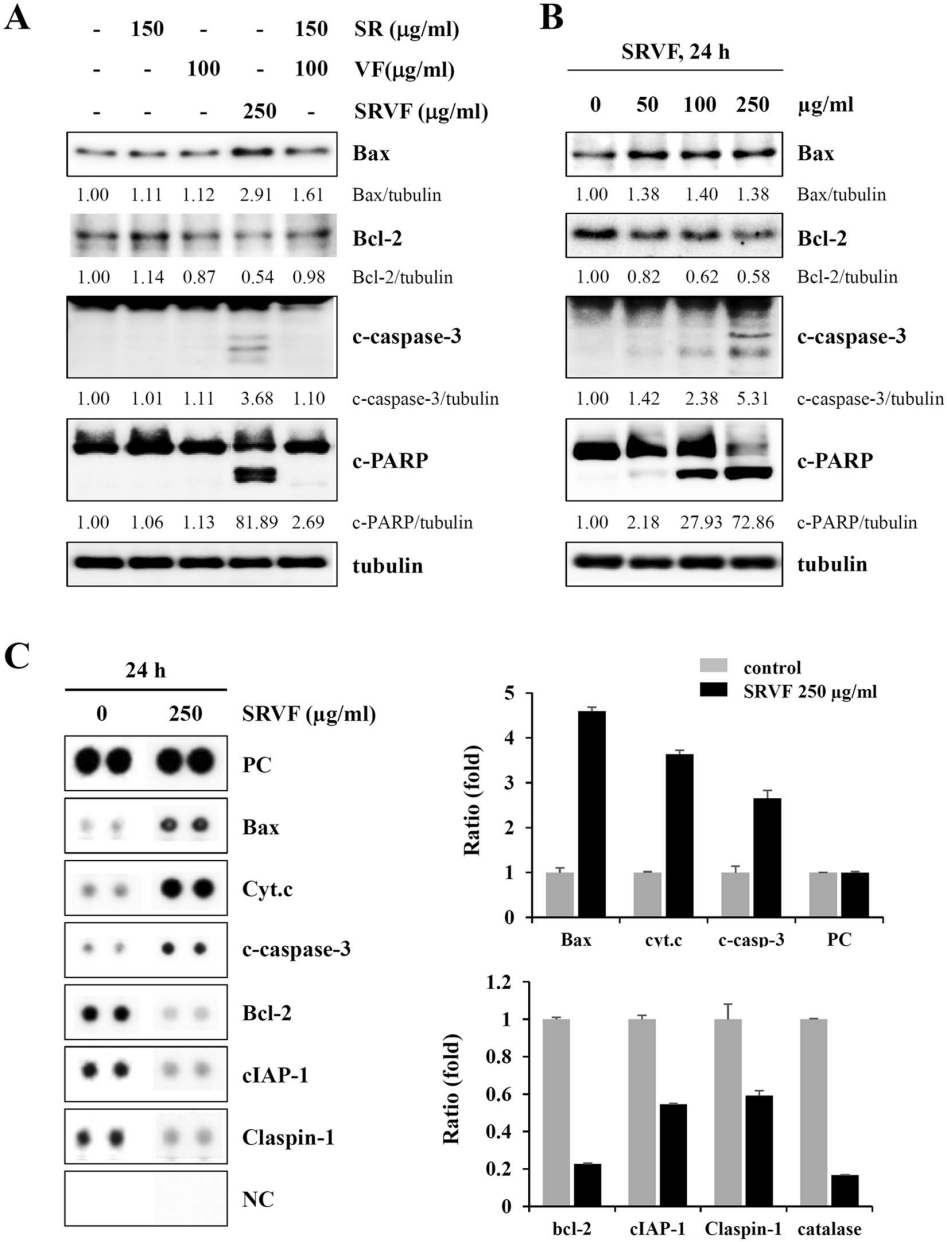
SRVF induces apoptosis in HT1080 cells. (**A**) Cells were treated with SR, VF, or SRVF at the indicated concentrations for 24 h and the levels of cell death-related proteins were examined by Western blot analysis. (**B**) Cells treated with increasing concentrations of SRVF for 24 h were analyzed for cell death-related proteins. Relative band intensities were calculated using ImageJ software after normalizing to tubulin expression. The full size blot is shown in Supplementary Fig. S4. (**C**) Levels of apoptosis-related proteins in SRVF-treated and –untreated HT1080 cells were determined using the Human Apoptosis Array Kit. Pixel densities were quantified using ImageJ software and the relative ratios compared to untreated control cells are expressed as the mean ± SD calculated from duplicate dots after normalizing to those of the positive control (PC) and negative control (NC).

Upon entering the cells, the acetoxymethyl ester group of calcein-AM (Live Green, component A) can be cleaved by intracellular esterase, yielding green fluorescence that is proportional to the number of live cells. Conversely, Dead Red (component B) enters unhealthy cells with reduced plasma membrane integrity and binds to intracellular DNA, resulting in red fluorescence that is proportional to the number of dead cells. As shown in Fig. 3 and Supplementary Fig. 3, SRVF-treated HT1080 cells and MDA-MB231 cells showed a significant increase in the number of dead cells and a concomitant decrease in the number of live cells in a dose-dependent manner, but not in cells treated with SR, VF, or their combination, consistent with the results from the CCK assay as well as cell cycle analysis. Western blot analysis revealed that SRVF treatment considerably up-regulated the levels of pro-apoptotic proteins Bax, cleaved caspase-3, and cleaved PARP, and down-regulated the level of the anti-apoptotic protein Bcl-2 in a dose-dependent manner (Fig. 4A,B). However, changes in the levels of these proteins by SR, VF, or their combination were insignificant compared to those induced by SRVF. In addition, results obtained from the Human Apoptosis Array Kit also confirmed that SRVF significantly increased the expression of pro-apoptotic proteins, including Bax, cytochrome c, and cleaved caspase-3, and decreased that of anti-apoptotic proteins, including Bcl-2, cIAP-1, and Claspin-1 (Fig. 4C).

### SRVF activated MAPK and inactivated STAT3 signaling in cancer cells

**Figure 5:**
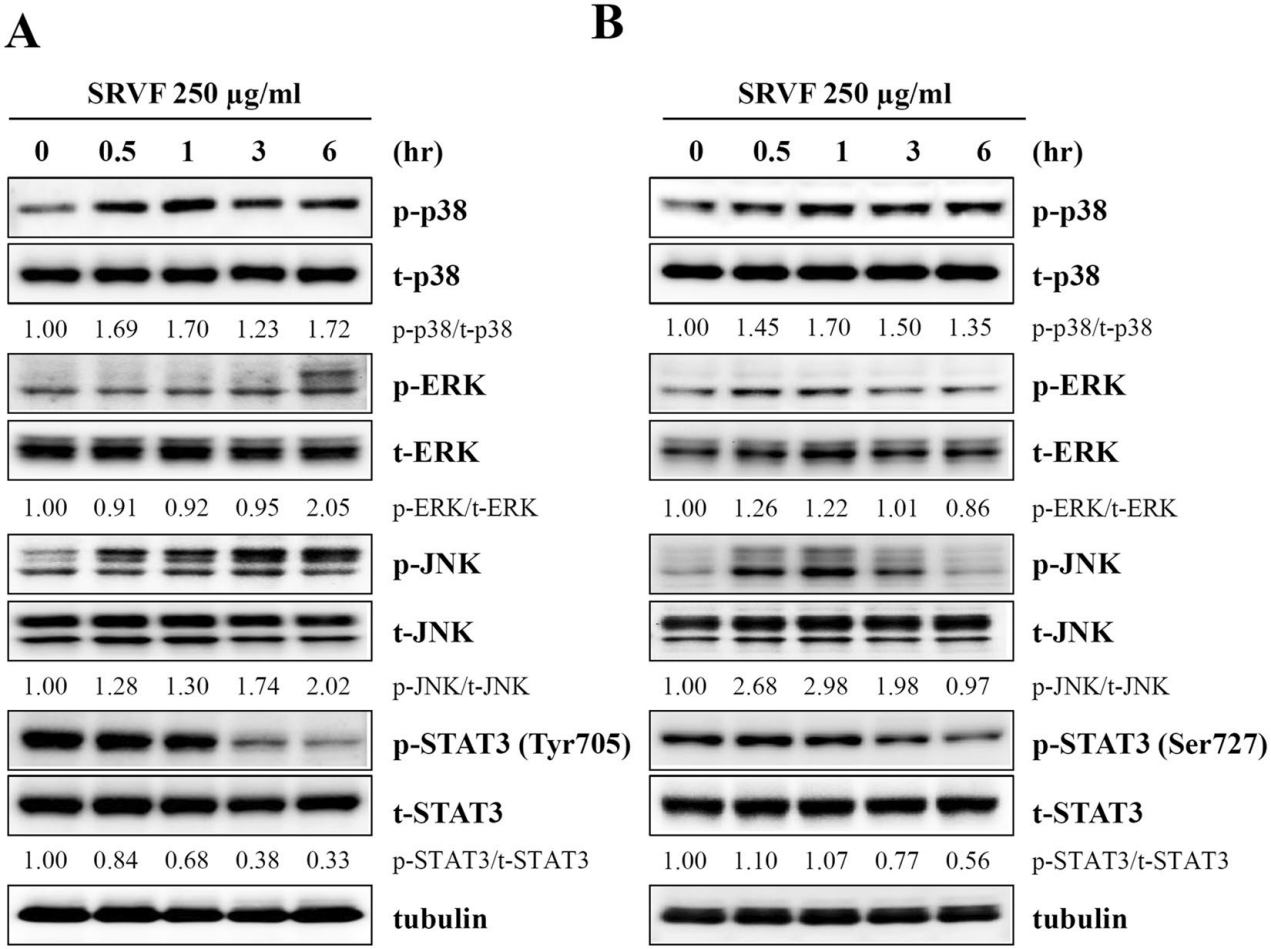
SRVF increases the phosphorylation of MAPK and decreases that of STAT3 in cancer cells. (**A,B**) HT1080 (**A**) and MDA-MB231 (**B**) cells were treated with 250 μg/ml SRVF for 0.5, 1, 3, or 6 h, and the levels of total and phosphorylated p38, ERK, JNK, and STAT3 were determined by Western blotting. Data are representative of two independent experiments and band intensities were calculated using ImageJ software after normalizing to tubulin expression. The full size blot is shown in Supplementary Fig. S5.

Previous studies have demonstrated that MAPK family members, including p38, ERK, and JNK, are crucial for the maintenance of cell growth^23,24^. In addition, constitutive activation of STAT3 in cancer cells induces cell transformation *via* the inhibition of apoptosis and cell cycle activation^25,26^. Therefore, interfering with the STAT3 pathway may serve as a therapeutic regimen for controlling cancer^27,28^. Western blot analysis showed that treatment with 250 µg/ml SRVF increased the levels of phosphorylated p38, ERK, and JNK in HT1080 (Fig. 5A) and MDA-MB231 (Fig. 5B) cells. In contrast, SRVF considerably suppressed the phosphorylation of STAT3, indicating that SRVF induced death of cancer cells by MAPK activation and STAT3 inactivation. In Hs27 normal human fibroblast cells, SRVF did not show greater effects compared with SR, VF, or its combination in terms of morphological changes and increase in the cell cycle- and apoptosis-related proteins. In addition, SRVF did not induce MAPK activation and suppress STAT3 phosphorylation (Supplementary Fig. 6).

### SRVF disrupted focal adhesion and F-actin organization

**Figure 6:**
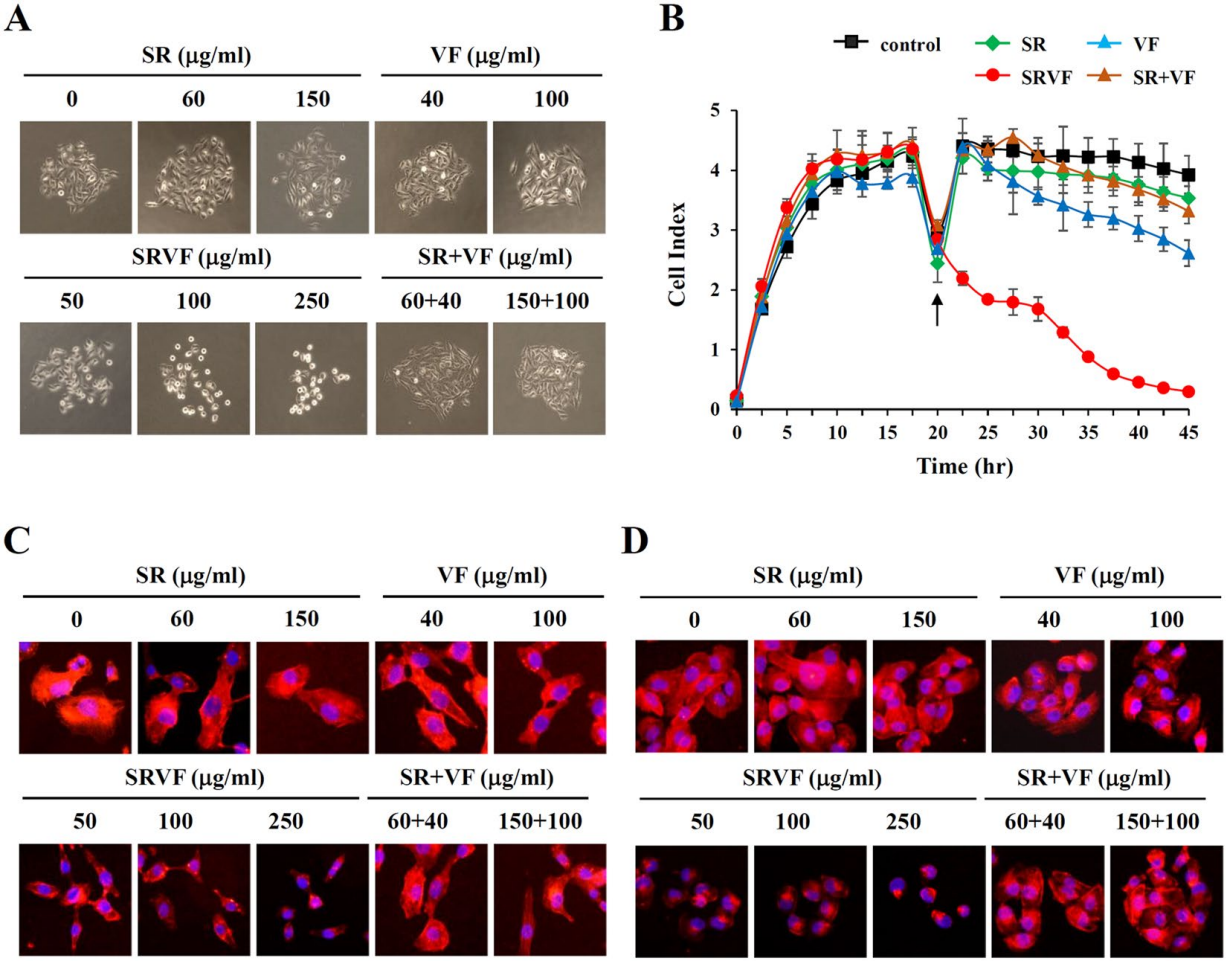
SRVF reduces cell adhesion and F-actin intensity in cancer cells. (**A**) Cancer cell colonies were treated with the indicated concentrations of SR, VF, or SRVF for 24 h and the morphological changes were observed under an inverted microscope. (**B**) Cells were seeded onto the E-Plate 16 and incubated in culture medium for 20 h. After replacing with fresh medium containing SR, VF, or SRVF, cells were monitored for electrical impedance for an additional 24 h. Data are expressed as the mean ± SD of triplicate samples. (**C**,**D**) HT1080 (**C**) and MDA-MB231 (**D**) cells were seeded onto the coverslips and treated with the indicated concentrations of SR, VF, or SRVF for 6 h and stained with TRITC-conjugated phalloidin. Nuclei were counterstained with DAPI and visualized under a fluorescence microscope. Data are representative of three independent experiments.

Anoikis, which is triggered by cell detachment and reorganization of the cytoskeleton, affects cell fate decision and suppresses metastatic spreading from the primary site and other organs^5^. Since SRVF caused the specific mode of cell death termed anoikis, we examined, in detail, the effects of SRVF on the loss of cell adhesion and disruption of the F-actin cytoskeleton. To mimic the colonization of cancer cells, a colony of hundreds of cells was formed from a single cell, which was then treated with SR, VF, or SRVF. As shown in Fig. 6A, SRVF-treated cells showed a loosening of interactions with neighboring cells and the surrounding ECM and detachment-induced cell death in a dose-dependent manner, whereas SR, VF, or their combination did not weaken intercellular adhesion. In addition, cells grown to confluency on the E-Plate 16 were also more efficiently detached by SRVF (Fig. 6B). Since it is widely appreciated that the F-actin cytoskeleton is essential for the generation of mechanical force and the regulation of cellular morphology^29,30^, we investigated changes in the F-actin cytoskeleton by staining with TRITC-phalloidin in cells treated with SR, VF, or SRVF. As shown in Fig. 6C, F-actin filaments were homogenously distributed throughout the cytoplasm in untreated control HT1080 cells. In contrast, the F-actin filaments in SRVF-treated cells were reorganized (a decrease in stress fiber formation and an increase in cortical actin formation), which caused the cells to assume a round shape. Similar effects were also observed in MDA-MB231 cells (Fig. 6D), indicating that SRVF significantly decreases adhesive activity by affecting F-actin organization.

### SRVF down-regulated the expression of focal adhesion-related proteins

**Figure 7:**
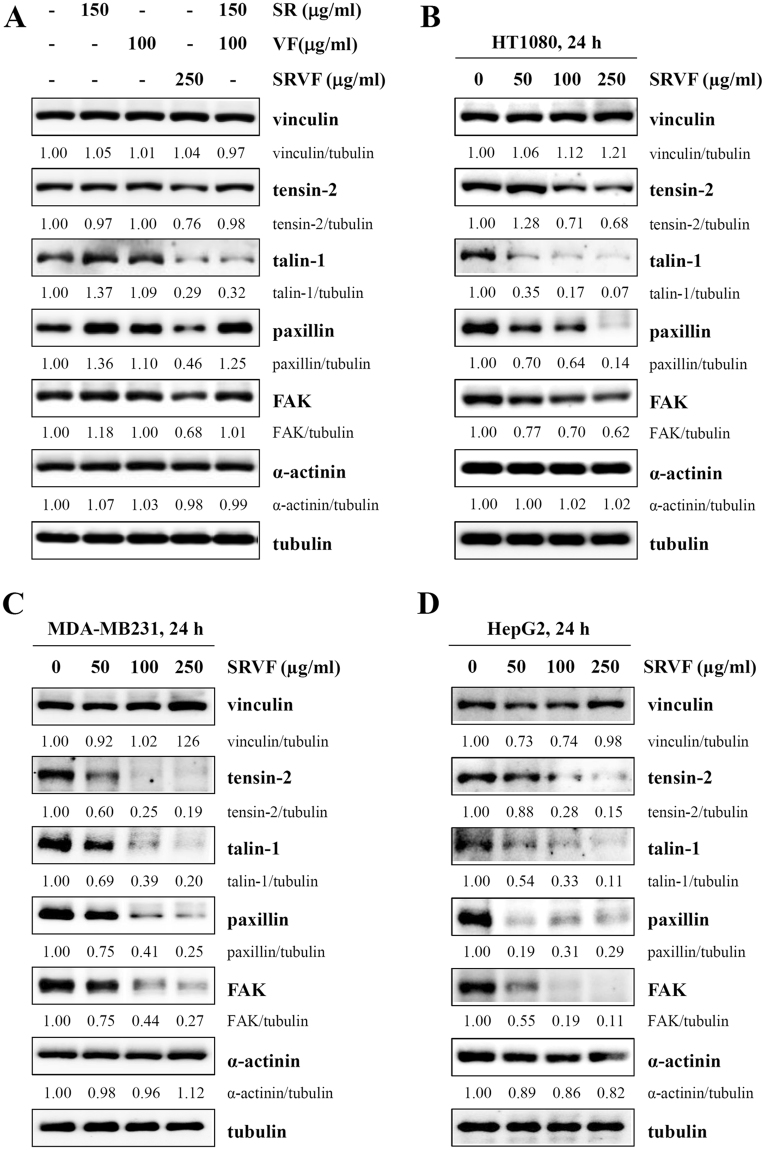
SRVF regulates the expression of focal adhesion-related proteins in cancer cells. (**A**) HT1080 cells were treated with SR, VF, or SRVF for 24 h, and total cell lysates were subjected to Western blotting. (**B–D**) HT1080, MDA-MB231, and HepG2 cells were treated with 50, 100, or 250 µg/ml SRVF for 24 h, and the levels of focal adhesion-related proteins were determined by Western blotting. Band intensities were calculated using ImageJ software after normalizing to tubulin expression. Data are representative of two independent experiments. The full size blot is shown in Supplementary Figs S7 and S8.

FAK is a widely expressed cytoplasmic protein tyrosine kinase that plays an essential role in the modulation of cell adhesion, migration, proliferation, and survival^31,32^. FAK is overexpressed in a number of human cancers, and the inhibition of FAK leads to the loss of adhesion and the induction of apoptosis in cancer cells^33–35^. During the process of cellular adhesion, cytoskeletal proteins, such as FAK, vinculin, tensin, talin, paxillin, and actinin, are recruited to the focal adhesion in an ordered and sequential manner, and are involved in actin polymerization^36,37^. Since SRVF disrupted actin organization and reduced adhesion to the ECM or neighboring cells, we next examined the levels of focal adhesion proteins by Western blot analysis. As shown in Fig. 7A,B, the levels of tensin-2, talin-1, paxillin, and FAK were significantly reduced in a dose-dependent manner following treatment with SRVF, while changes in the levels of vinculin and α-actinin were insignificant. In contrast, treatment with SR or VF alone caused only a slight effect on the expression of focal adhesion proteins. In addition, these inhibitory effects caused by SRVF were confirmed in other cancer cells, including MDA-MB231 and HepG2 cells (Fig. 7C,D). These data collectively indicate that SRVF causes detachment-induced apoptosis *via* down-regulating focal adhesion of cancer cells.

### Oral administration of SRVF suppressed tumor growth *in vivo* with no adverse effects

**Figure 8:**
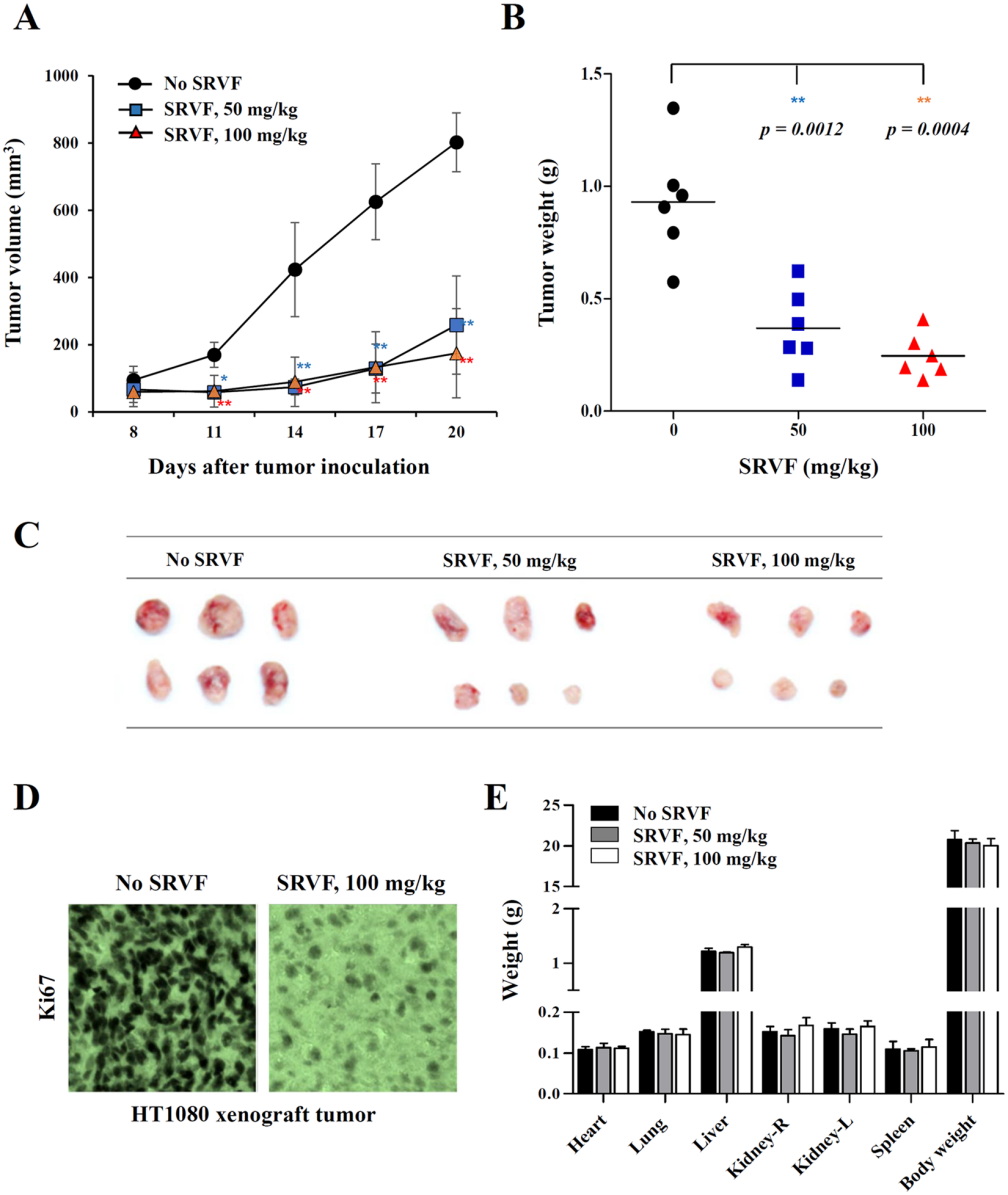
SRVF administration suppresses tumor growth *in vivo*. Balb/c nude mice were subcutaneously injected with HT1080 cells. After 7 days, mice were administered an equal volume of saline (control) or 50 or 100 mg/kg SRVF daily for 14 days (n = 6 per group). (**A**) Tumor volume was calculated by measuring the tumor size on two axes of the tumors according to the following formula: tumor volume = (length) × (width)^2^ × 0.52. Statistical significance was evaluated with Student’s *t*-test. *p < 0.05, **p < 0.01 vs. No SRVF. (**B**,**C**) At the time of sacrifice (day 21), tumors were excised, weighed, and photographed. Bars represent the mean value of each group (n = 6), and statistical significance was determined using Student’s t-test. Statistical significance was evaluated with Student’s *t*-test. **p < 0.01 vs. No SRVF. (**D**) Cryosections of HT1080 xenograft tumors obtained from saline-treated control group and SRVF-treated group were immunostained for Ki67 expression. (**E**) Each group of normal mice (n = 3) was administered saline or, 50 or 100 mg/kg SRVF for 21 days. After sacrifice, body and organ weight were measured and are expressed as the mean ± SD.

To evaluate the inhibitory effect of SRVF on tumor growth *in vivo*, Balb/c nude mice with a tumor mass were administered saline or SRVF daily for 14 days. As shown in Fig. 8A, treatment with 50 or 100 mg/kg SRVF efficiently suppressed tumor growth, resulting in a decrease in tumor volume by approximately 67.8% and 78.2%, respectively, compared with saline treatment on day 20 (F = 44.79, *p* < 0.0001, one-way ANOVA). The tumor weights of saline-treated control mice were 0.93 ± 0.26 g, while those treated with 50 or 100 mg/kg SRVF were 0.37 ± 0.17 g and 0.25 ± 0.11 g, respectively, reflecting a reduction of 60.2% and 73.1%, respectively (F = 20.40, *p* < 0.0001, one-way ANOVA) (Fig. 8B,C). Immunohistochemical staining of HT1080 xenograft sections showed that cell proliferation marker Ki67 in tumors treated with SRVF was significantly decreased in comparison with that in saline-treated control tumors (Fig. 8D), indicating that SRVF suppressed *in vivo* cell proliferation. To further confirm the safety of SRVF *in vivo*, organ weight and body weight were measured at the time of sacrifice after daily oral administration of SRVF (50 or 100 mg/kg) or saline to normal mice with no tumors for 21 days. As shown in Fig. 8E, SRVF-treated mice showed similar levels in organ weight and body weight compared with those of saline-treated control mice, indicating that SRVF retarded tumor growth with no adverse effects.

## Discussion

Over the last decade, the single-drug-based chemotherapy paradigm in the treatment of chronic diseases, including cancer, diabetes, atherosclerosis, and inflammation, has gradually transitioned to multi-drug chemotherapy, due to low effectiveness, drug resistance, and adverse effects of the single-drug paradigm. Many diseases, including cancer, are not caused by a single gene or a single pathway that has mutated or been altered among patients^38^. Therefore, interest in decoctions of herbal mixtures has increased with the goal of pursuing maximal therapeutic efficacy and minimal unwanted side effects, and that of improving patients’ overall status, under the assumption that the decoction works synergistically and/or influences several related pathways simultaneously. Previous studies on chronic diseases, such as cancer, diabetes mellitus, and hepatic disorders, have demonstrated that a decoction from a combination of herbs is more beneficial to patients than treatment from a single herb^12,39^. The antagonistic effect of herbal mixtures has also been reported; therefore, finding optimal herbal combinations and determining their optimal composition ratio and/or dose are critical for the development of new drugs using herbal medicine^40,41^.

In this study, we intended to prove the superior anti-cancer efficacy and detailed mechanism of action of SRVF, a novel herbal decoction including SR and VF, by means of a comparison with those of SR, VF, or their co-treatment. SR derived from *Scrophulariae ningpoensis* has long been used as a traditional Chinese medicine to treat pyrexia with impairment of yin by eliminating heat and replenishing vital essence. Recent studies have demonstrated that the ethanolic extract of SR alleviates ventricular remodeling by modulating cardiac hypertrophy-associated factors and may have therapeutic potential to postpone heart failure caused by cardiovascular diseases, such as myocardial infarction and hypertension^15^. It has been also reported that SR inhibits the production of inflammatory cytokines in lipopolysaccharide-activated Raw 264.7 cells, decreases the symptoms of 2,4-dinitrochlorobenzene-induced allergic contact dermatitis in mice, and suppresses hypoxia-induced neurotoxicity by scavenging hypoxia-induced inflammatory cytokines, inhibiting the activation of NF-κB, and reducing HIF-1α generation in microglial cells^14^. However, the anti-cancer efficacy of SR has not yet been reported, consistent with the fact that there is little cytotoxicity following treatment with SR up to 1,000 µg/ml; indeed, cell proliferation was increased in both cancer and normal cells (Fig. 1). However, VF contains a large amount of flavonoids, including casticin, apigenin, isoorientin, hesperidin, and isovitexin, which exhibit anti-tumor activities^19,20,42^. For example, casticin inhibited the proliferation and induced G_2_/M cell cycle arrest in human cervical cancer HeLa cells and caused caspase-induced apoptosis in human lung and colon cancer cells^19,43,44^. In this study, we also observed cytotoxic effects of VF water extract on human gastric cancer cells (AGS, IC_50_ = 482.5 µg/ml), human ovarian cancer cells (SK-OV-3, IC_50_ = 527.6 µg/ml), and human fibrosarcoma cells (HT1080, IC_50_ = 610.3 µg/ml) (data not shown), although the cytotoxicity was very low or not observed at concentrations lower than 100 µg/ml. The effects of co-treatment with 150 µg/ml SR and 100 µg/ml VF were similar to the sum of each one and anti-cancer activity was insignificant. However, SRVF induced dramatic changes in cellular morphology and caused a remarkably stronger cytotoxic effect in malignant cancer cells than did that of SR, VF, or their co-treatment (Fig. 1). SRVF increased the cell population in G_2_/M phase at 12 h and in apoptotic subG_0_/G_1_ phase at 24 h by modulating cell cycle- and apoptosis-related proteins (Figs 2–4). In particular, SRVF significantly reduced focal adhesion and F-actin organization by down-regulating focal adhesion-related proteins (Figs 6–7), which resulted in detachment-induced apoptosis, indicating that SRVF exhibits anti-cancer efficacy by restoring the anoikis sensitivity of cancer cells. The *in vivo* xenograft experiment revealed that SRVF administration at doses of 50 mg/kg and 100 mg/kg considerably suppressed tumor growth by approximately 70–80% without affecting body or organ weight (Fig. 8). Furthermore, daily treatment with 10 mg/kg SRVF efficiently inhibited tumor growth by approximately 50% (Supplementary Fig. 9). We also observed that VF administration inhibited tumor growth and decreased tumor weight by approximately 30.12%, compared with saline treatment, whereas SR and SR + VF treatment did not suppress tumor growth (Supplementary Fig. 10).

It is believed that normal epithelial cells must attach to the ECM to survive and, when cells lose cell-matrix adhesion, they rapidly undergo a form of apoptosis known as anoikis^3,5^. In contrast, during tumor progression, cells acquire resistance to anoikis, which enables tumor cells to survive after separating from the ECM and allows them to proliferate and colonize at a distant secondary site, conferring increased dissemination *in vivo* ^4,7^. In suspension, cancer cells survive and form aggregates in which cell-cell contacts are maintained *via* bypassing anoikis. Since SRVF significantly reduced cell adhesion to the ECM and caused detachment-induced apoptosis under adherent culture conditions, we next examined whether SRVF affected the survival and/or formation of cell aggregates in suspension. As shown in Supplementary Fig. 11, MDA-MB231 cells rapidly proliferated as cell aggregates in suspension, while SRVF-treated MDA-MB231 cells did not form cell aggregates. Moreover, the interactions between cells were weak and scattered, confirming that SRVF efficiently overcomes anoikis resistance, which is important for preventing proliferation as well as the metastatic spread of malignant cancer cells. In fact, numerous studies have demonstrated that the inhibition of cell aggregate formation leads to anoikis, and cell aggregation confers multi-drug resistance in malignant cancer cells^45–48^. Therefore, targeting anoikis resistance and cell aggregation represents a promising strategy for anti-cancer therapy.

In summary, we demonstrated that SRVF efficiently causes detachment-induced apoptosis of malignant cells by disrupting cell-cell and cell-ECM interactions, down-regulating focal adhesion-related proteins, and reorganizing the F-actin cytoskeleton. Furthermore, we observed the therapeutic efficacy of SRVF against tumor growth *in vivo* using a xenograft model, suggesting the potential of SRVF as an anti-cancer agent for the treatment of malignant cancer cells.

## Electronic supplementary material

Supplementary information
