# Supplementary material for: SRVF, a novel herbal formula including Scrophulariae Radix and Viticis Fructus, disrupts focal adhesion and causes detachment-induced apoptosis in malignant cancer cells
Source: Sci Rep. 2017 Oct 16;7:12756. doi: 10.1038/s41598-017-12934-y (PMC5643381; doi:10.1038/s41598-017-12934-y)
Supplement: Supplementary file 1 — Supplementary information [file 41598_2017_12934_MOESM1_ESM.pdf]

Supplementary Information

SRVF, a novel herbal formula including *Scrophulariae Radix* and *Viticis Fructus*, disrupts focal adhesion and causes detachment-induced cancer cell death

Aeyung Kim, Minju Im and Jin Yeul Ma*

Korean Medicine (KM) Application Center, Korea Institute of Oriental Medicine (KIOM), 70 Chumdan-ro, Dong-gu, Daegu 701-300, Republic of Korea

- Supplementary Figure S1
- Supplementary Figure S2
- Supplementary Figure S3
- Supplementary Figure S4
- Supplementary Figure S5
- Supplementary Figure S6
- Supplementary Figure S7
- Supplementary Figure S8
- Supplementary Figure S9
- Supplementary Figure S10
- Supplementary Figure S11

*Correspondence and requests for materials should be addressed to J.Y.M (jyma@kiom.re.kr)

Figure S1

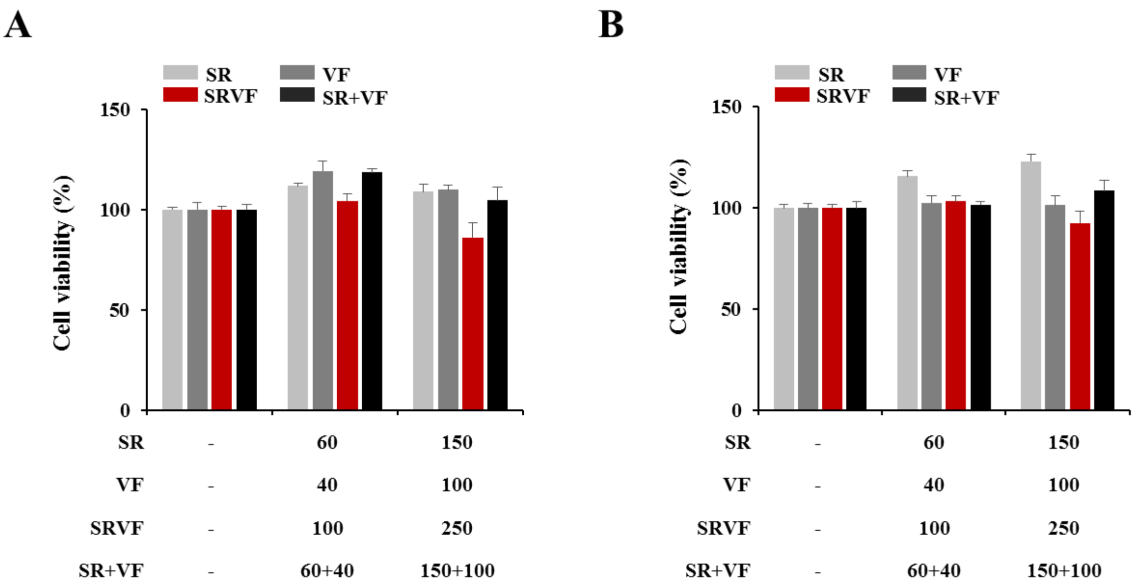

Figure S1. SRVF did not significantly reduce the cell viability in normal cells. (A-B) Hs27 (A) and MCF-10A (B) cells were incubated with the indicated doses of SR, VF, or SRVF at concentrations of 50, 100, or 250 μg/ml. After 24 h, cell viability was determined using the CCK assay and is expressed as the mean ± standard deviation (SD).

Figure S2

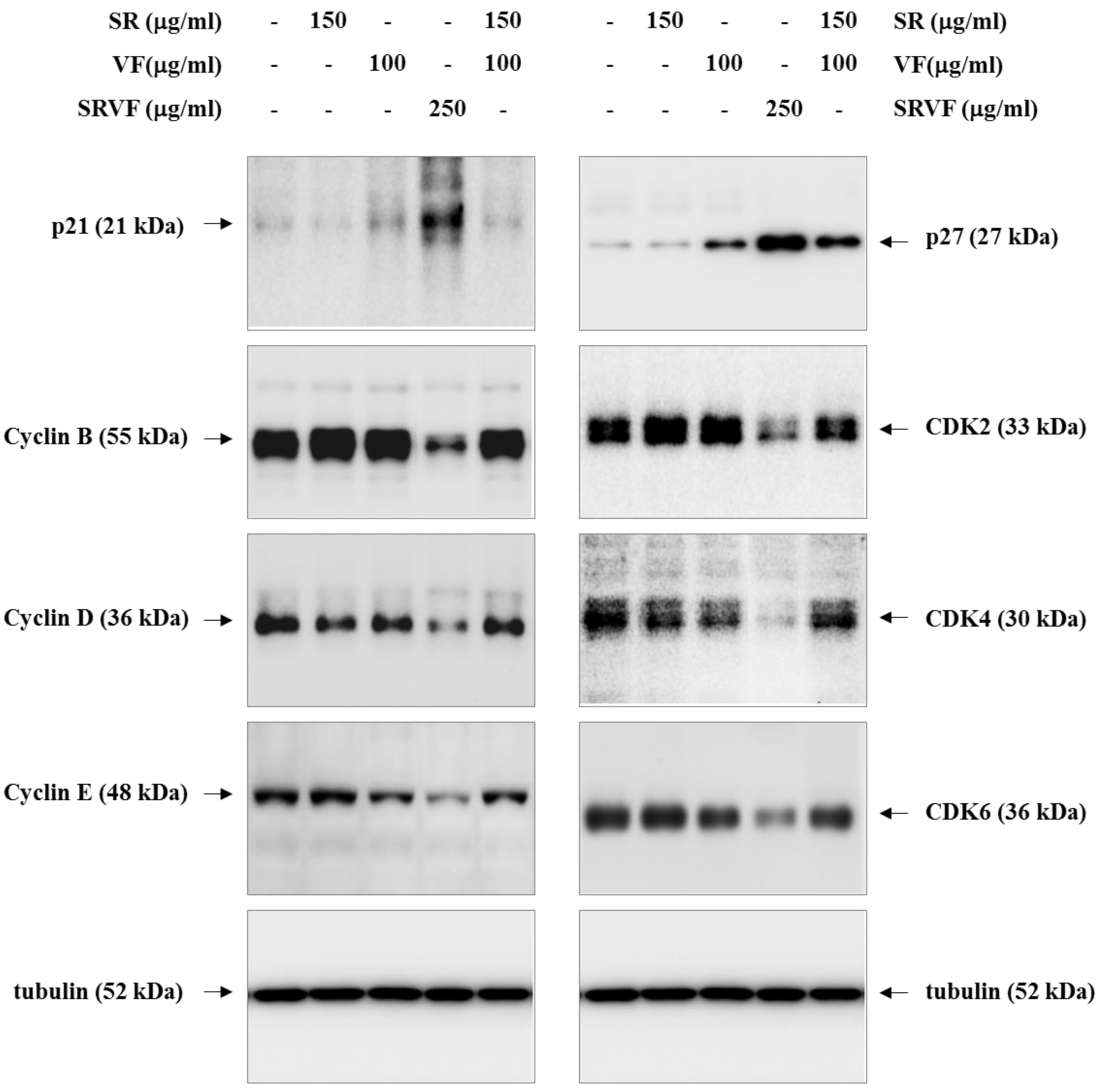

Figure S2. SRVF regulates cell cycle-related proteins in HT1080 cells. This is a full length image of the cropped blots presented in the Figure 2B.

Figure S3

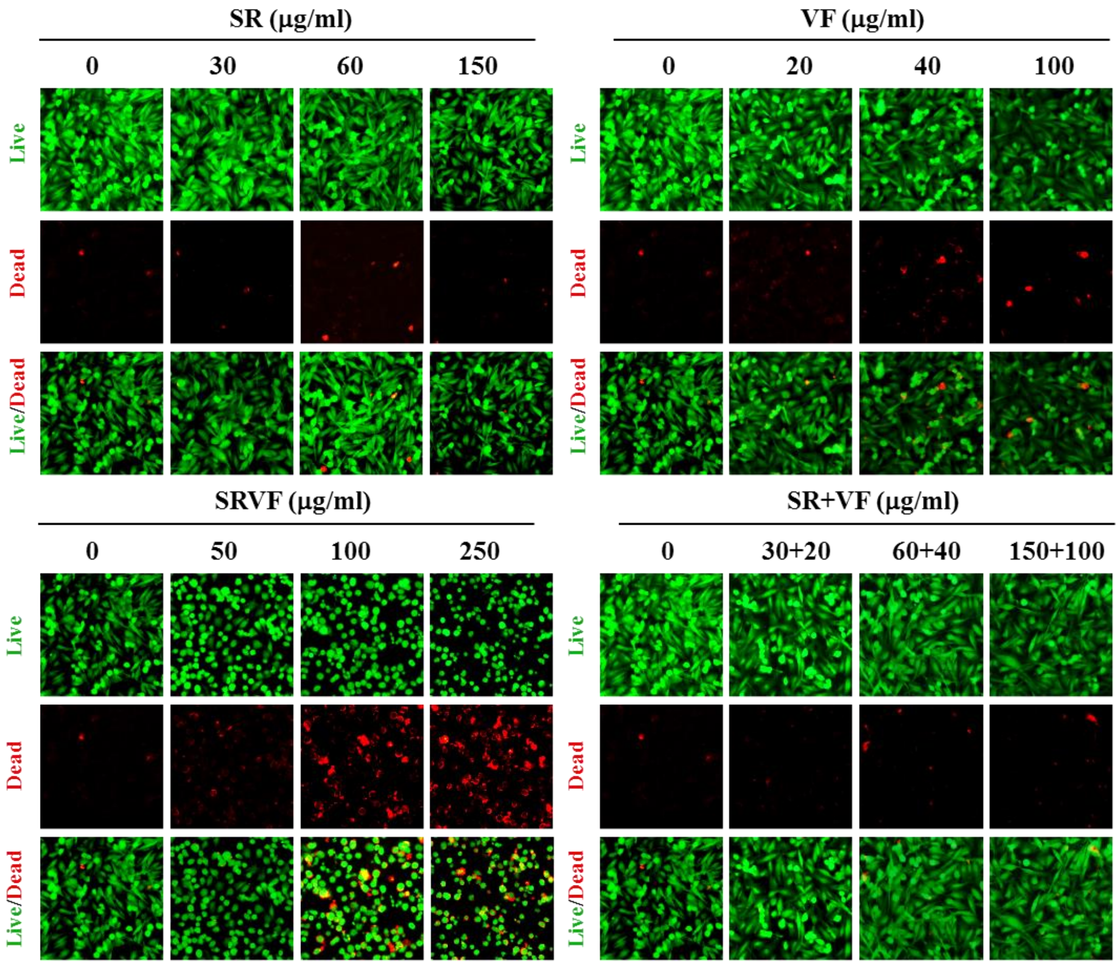

Figure S3. SRVF induces severe cytotoxicity in MDA-MB231. Cells were seeded onto the 24-well culture plates and incubated for 12 h to adhere. After incubating cells with the indicated doses of SR, VF, or SRVF for 24 h, live and dead cells were labeled using the LIVE/DEAD Cell Imaging Kit and observed under a fluorescence microscope.

Figure S4

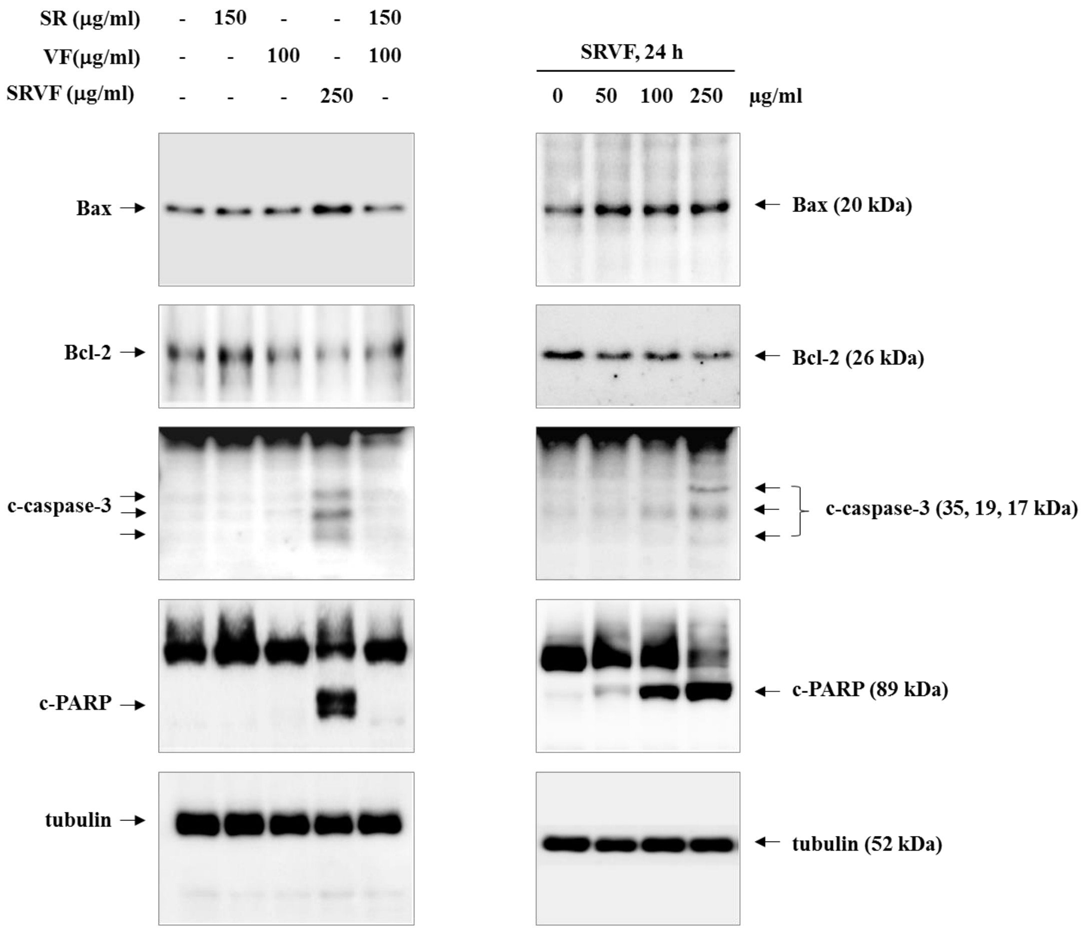

Figure S4. SRVF induces apoptotic cell death in HT1080 cells. This is a full length image of the cropped blots presented in the Figure 4A and 4B.

Figure S5

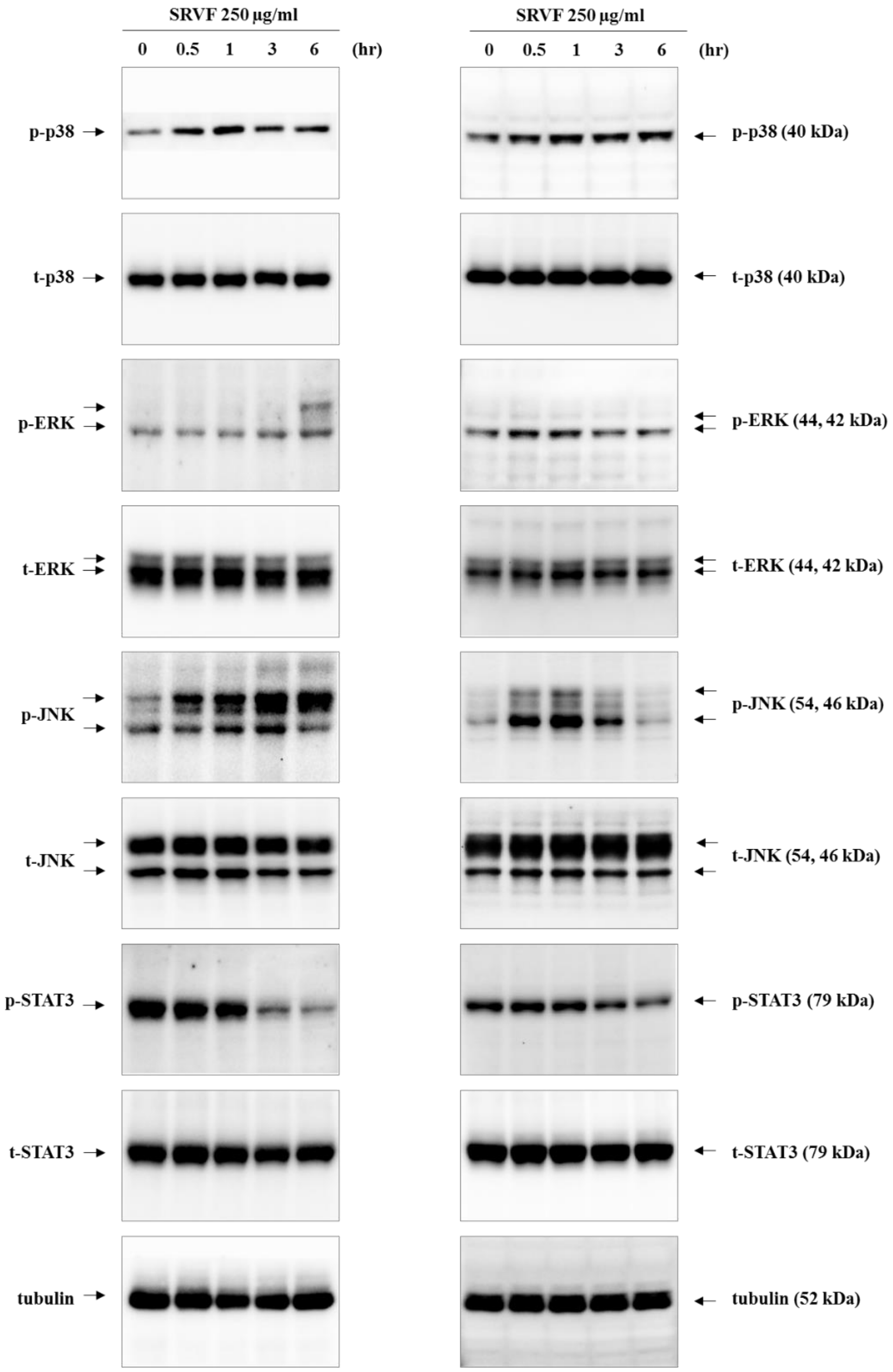

Figure S5. SRVF increases phosphorylation of MAPK and decreases phosphorylation of STAT3 in cancer cells. This is a full length image of the cropped blots presented in the Figure 5A and 5B.

Figure S6

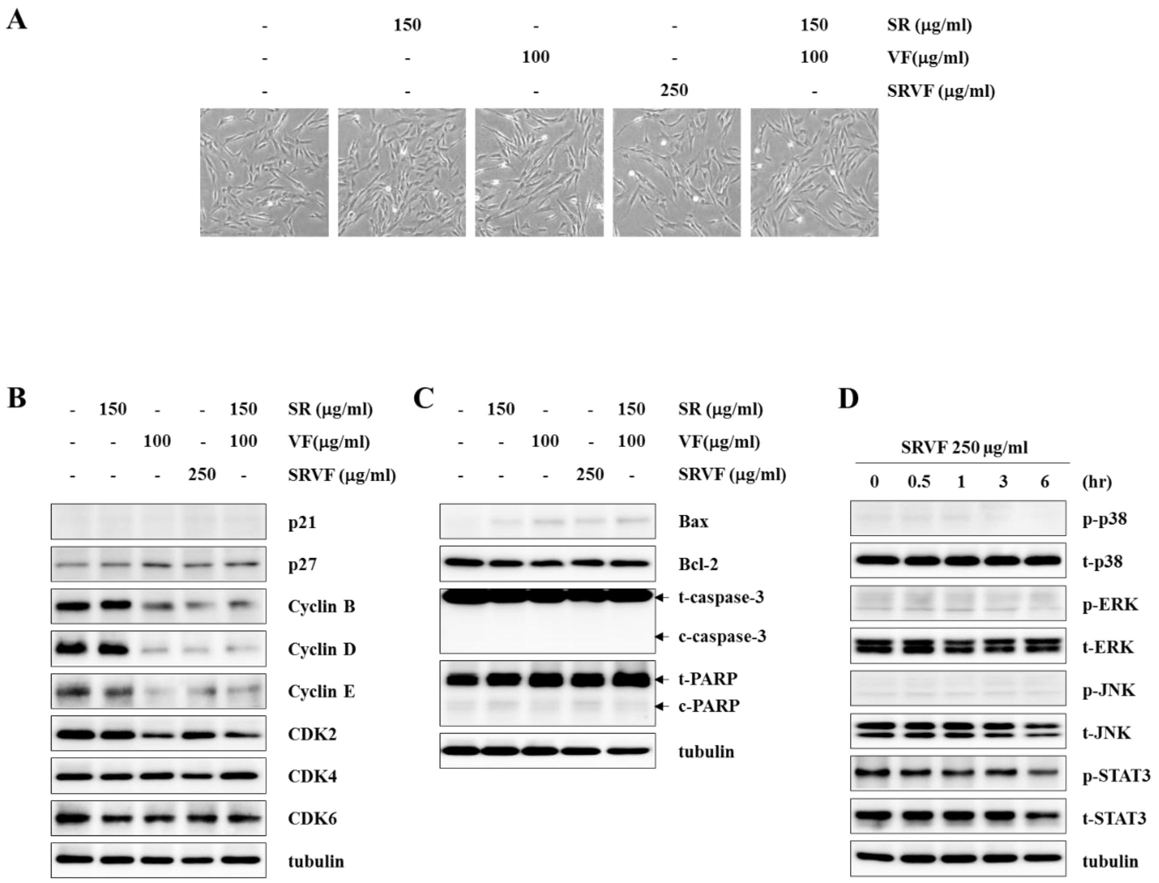

Figure S6. The effect of SRVF on the Hs27 human fibroblast cells. (A) Hs27 cells were incubated with the indicated concentrations of SR, VF, or SRVF for 24 h and photographed. **(B-C)** Cells were incubated with indicated concentration of SR, VF, or SRVF for 12 h (B) or 24 h (C), and then the levels of cell cycle-related proteins and apoptosis-related proteins were examined by Western blot analysis, respectively. **(D)** Hs27 cells were treated with 250 µg/ml SRVF for 0.5, 1, 3, or 6 h, and the levels of total and phosphorylated MAPKs and STAT3 were detected by Western blotting.

Figure S7

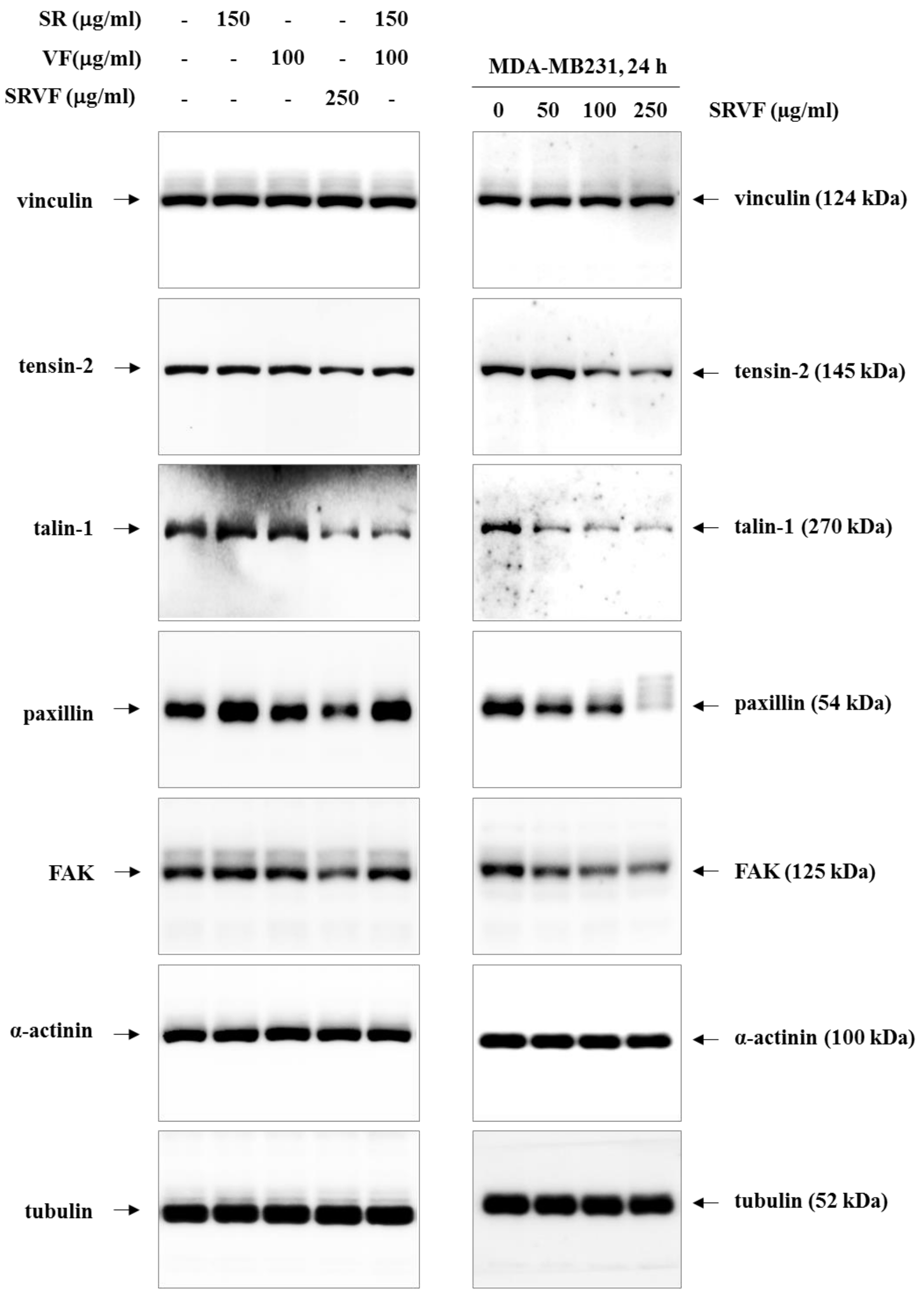

Figure S7. SRVF regulates the expression of focal adhesion-related proteins in HT1080 cells. This is a full length image of the cropped blots presented in the Figure 7A and 7B.

Figure S8

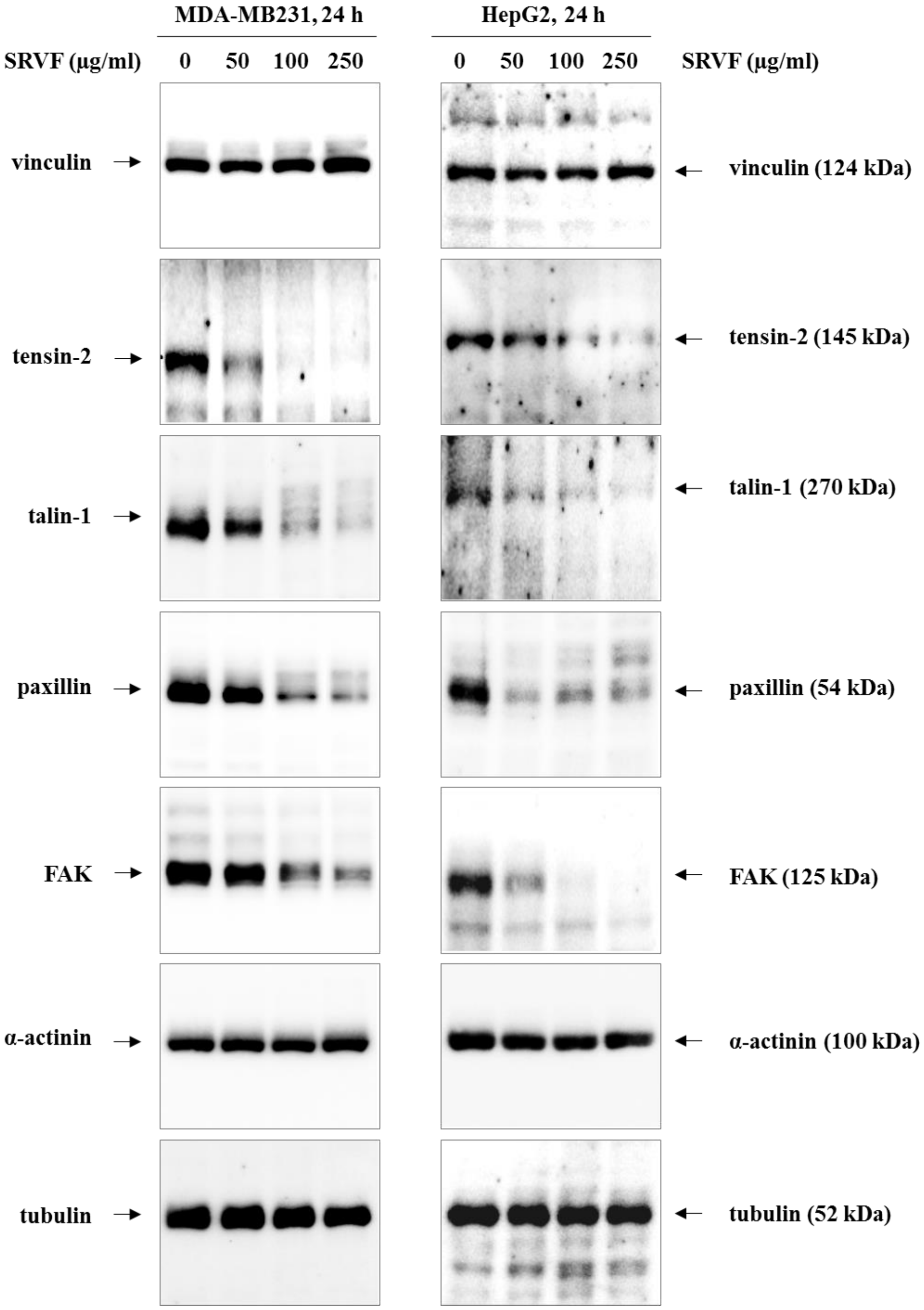

Figure S8. SRVF regulates the expression of focal adhesion-related proteins in MDA-MB231 and HepG2 cells. This is a full length image of the cropped blots presented in the Figure 7C and 7D.

Figure S9

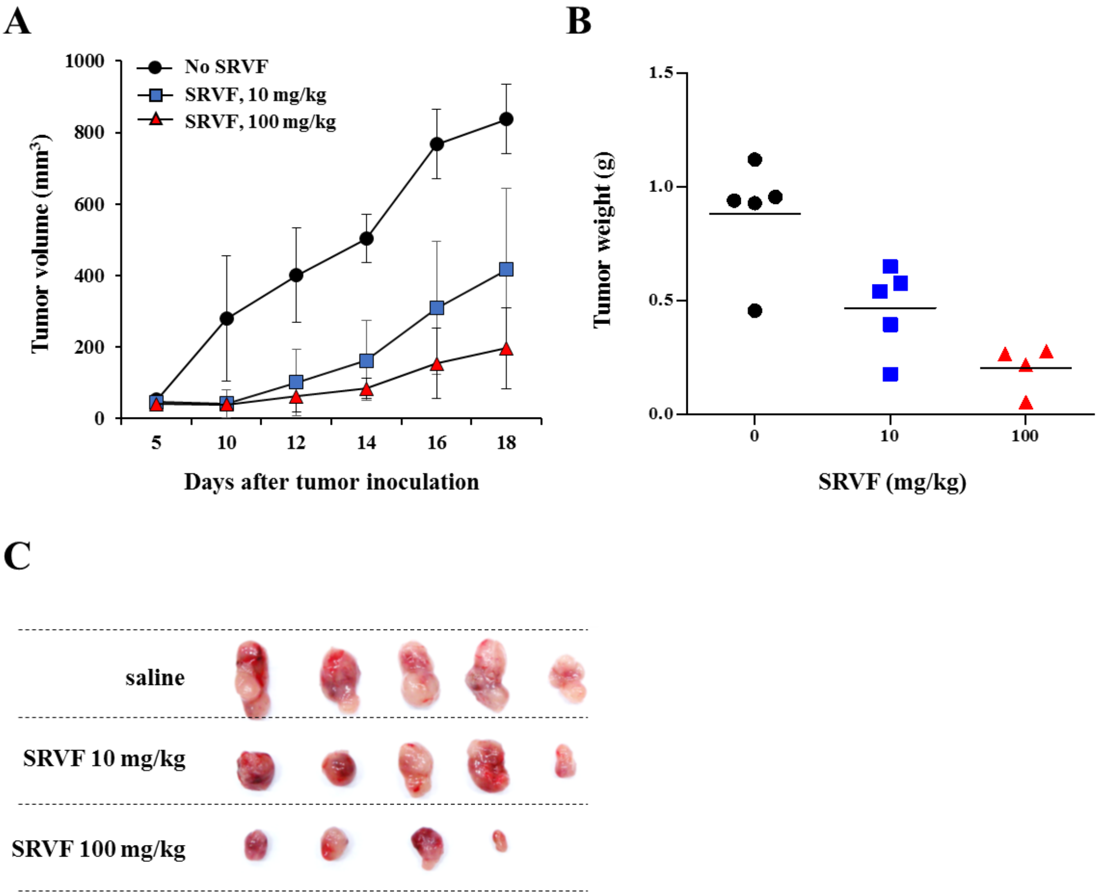

Figure S9. SRVF administration at 10 mg/kg suppresses tumor growth *in vivo*. Balb/c nude mice were subcutaneously injected with HT1080 cells (2×10^6 cell/200 µl PBS). After 5 days, mice were divided into each group (n = 4 or 5 per group) and daily administered an equal volume of saline (control), 10 mg/kg or 100 mg/kg SRVF for 13 days. **(A)** Tumor volume was calculated by measuring the tumor size on two axes of the tumors. **(B)** At the time of sacrifice (day 18), tumors were excised and weighed. Bars represent the mean value of each group. **(C)** Excised tumors were photographed.

Figure S10

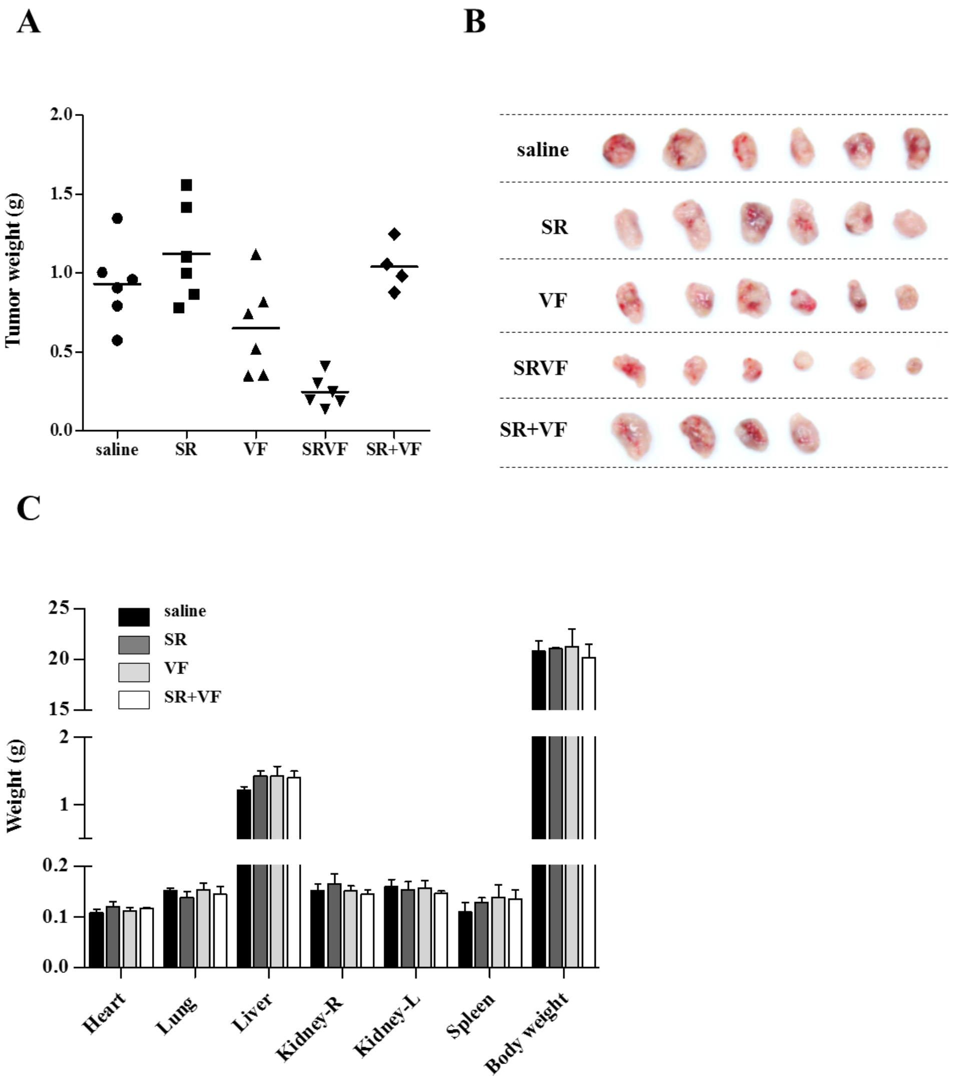

Figure S10. SRVF and VF administration suppresses tumor growth *in vivo*. (A) Balb/c nude mice were subcutaneously injected with HT1080 cells (2×10^{6} cell/200 µl PBS). After 7 days, mice were divided into each group (n = 4 or 6 per group) and daily administered an equal volume of saline (control), 60 mg/kg SR, 40 mg/kg VF, or 100 mg/kg SRVF for 14 days. At the time of sacrifice (day 21), tumors were excised and weighed. Bars represent the mean value of each group. **(B)** Excised tumors were photographed. **(C)** Normal mice with no tumor were administered an equal volume of saline (control), 60 mg/kg SR, 40 mg/kg VF, or 100 mg/kg SRVF for 21 days. Body and organ weight were measured after sacrifice and are expressed as the mean ± SD.

Figure S11

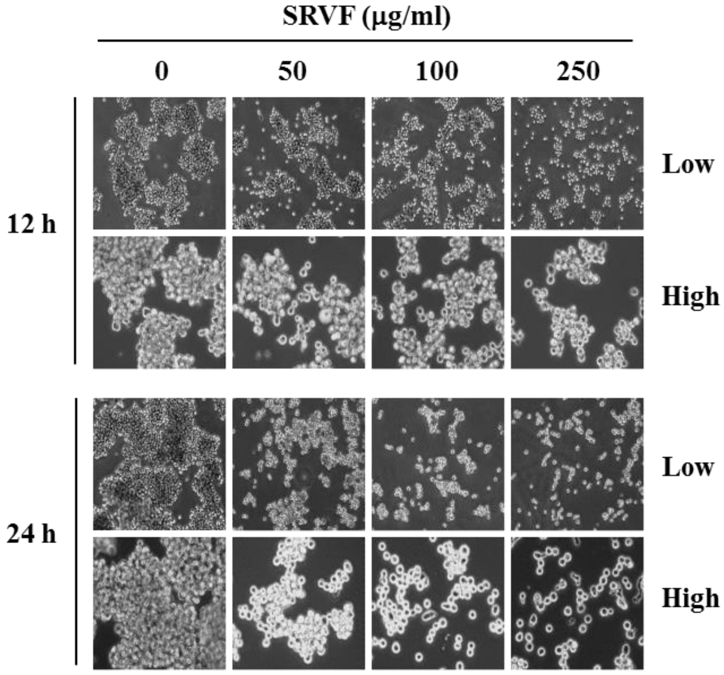

Figure S11. SRVF inhibits the formation of cell aggregates in suspension. MDA-MB231 cells suspended in DMEM/F12 media (Gibco,Carlsbad, CA, USA) containing 5% FBS, 10 ng/ml epidermal growth factor (EGF, Promokine, Heidelberg, Germany), 10 μg/ml insulin (Sigma), and 1 μg/ml hydrocortisone (Sigma) were seeded onto 6-well ultra-low adhesion plate (1×10^5 cell/well), treated with indicated concentrations of SRVF, and observed under an inverted microscope during 24 h.
